# Identification of Gene Coexpression Modules and Prognostic Genes Associated with Papillary Thyroid Cancer

**DOI:** 10.1155/2022/9025198

**Published:** 2022-09-20

**Authors:** Yanbing Shen, Wenfei He, Dan Wang, Ding Cao, Hongliang Mei, Tianji Luan, Yilin Hu

**Affiliations:** ^1^Department of General Surgery, General Hospital of Central Theater Command, Wuhan, Hubei 430070, China; ^2^Central Theater Command General Hospital and Hubei Key Laboratory of Central Nervous System Tumor and Intervention, Wuhan, Hubei 430070, China

## Abstract

Thyroid cancer is a great part of the endocrine tumor with an increasing incidence. Papillary thyroid carcinoma (PTC) is the most common subtype. With the enormous pace taken in the microarray technology, bioinformatics is applied in data mining more frequently. Weighted gene coexpression network analysis (WGCNA) can perform analysis combining clinic information. We performed WGCNA for prognostic genes associated with PTC. From the GEO profile, we got ten modules. We identified a key module that was closest to patients' survival time. Then, we screened five hub genes (ATRX, BOD1L1, CEP290, DCAF16, and NEK1) from the key module based on the clinical information from TCGA. These five genes not only significantly differ between the normal and tumor groups but have prognostic value. The receiver operating characteristic (ROC) curve indicated that they had the potential to serve as prognostic genes. We performed next-generation sequencing using the PTC tissue to get more convincing evidence. Besides, we established a new signature and verified it through K-M plots and ROC. The signature could be an independent factor for the prognosis of PTC, and we built a nomogram to carry out a quantitative study. In a word, the hub genes we explored in the study deserved more basic and clinical research.

## 1. Introduction

Thyroid cancer represents a series of carcinomas with an increasing incidence and high mortality [1, 2]. Thyroid cancer belongs to the malignant tumor of the endocrine system, and it locates in the head and neck. There are four common subtypes, papillary thyroid carcinoma, follicular thyroid carcinoma, undifferentiated carcinoma, and medullary thyroid carcinoma [3]. Papillary thyroid cancer (PTC) accounts for the most. The age at first diagnosis of PTC is decreasing. Moreover, PTC directly leads to 10%-15% of the death rate of patients. Early diagnosis of PTC seems arduous but paramount to decreasing morbidity and mortality.

Nowadays, several pieces of research are exploring potential biologic markers for the molecular pathogenesis of PTC. Nevertheless, few are introduced into clinical application. BRAF is a biological marker widely used in clinical work and serves as an indicator of mutation. Many evaluation indexes select BRAF as a standard to test the value of biological markers found in research. Finding more biologic markers like BRAF with clinical significance may facilitate the early diagnosis and treatment of PTC.

Currently, the full application of microarray technology and bioinformatics analysis can assist us in discovering new clues regarding hub genes [4–8]. However, traditional difference analysis has a deadly shortcoming, a lack of clinical information [9]. This point limits the application of bioinformatics analysis in clinical work. We use weighted gene coexpression network analysis (WGCNA) to enhance the value of the outcomes we got. WGCNA corresponds to an information reduction method and unsupervised classification strategy [10]. The advance of WGCNA lies in identifying gene modules and undergoing significant analysis with phenotypes. It is now universally applied to explore potential biomarkers for early cancer diagnosis, clearing the molecular mechanism of tumor development and targets for individual treatment.

In this passage, we selected three databases, one to get the prognostic genes and two for validation using. We focus on a series of phenotypes for hub genes. Furthermore, we compared the clinical model of hub genes we selected and BRAF to clarify this study's clinic value. Then, we supplemented the pathological section to enhance our conclusions' reliability.

## 2. Materials and Methods

### 2.1. Data Acquisition

Raw data are from Gene Expression Omnibus (http://www.ncbi.nlm.nih.gov), a communal dataset for genomics data of microarrays, chips, and high-throughput gene expression data submitted by the research community [11]. We screened the whole dataset and selected GSE60542 because it has enough samples and complete clinic information. GSE60542 uses the GPL570 platform (Affymetrix Human Genome U133 Plus 2.0 Array), including gene expression of 92 thyroid cancer samples. There are 61 PTC samples with high-quality information on pathology grade.

### 2.2. Data Procession

The data from GSE60542 have already been normalized. After probe summarization, 21151 genes were selected for further research. We listed all the genes' expression variance between PTC and healthy from GSE60542. Then, the top 20% of most different genes by analysis of variance (4230 genes) were selected as differentially expressed genes for the next WGCNA.

### 2.3. Gene Coexpression Network Construction

Firstly, we text the gene and sample profiles to ensure they are good genes and good samples. We found 8 outlier samples and excluded them. Secondly, we underwent the WGCNA work with the assistance of the “WGCNA” package in R software (3.6.3) [12], the steps we used as the ways that had been described previously [12].

### 2.4. Identified and Verified Hub Genes

The hub genes fit the following standard [13]: in the WGCNA module–trait relationships, (1) high within-module connectivity (cor.geneModuleMembership > 0.8) and (2) high correlation with certain clinical trait (cor.traitGeneSignificance > 0.2). In the PPI work, genes connect at less one gene. To assess the hub genes' clinic value, we use the data from The Cancer Genome Atlas Project database (TCGA, https://cancergenome.nih.gov/). The Human Protein Atlas (http://www.proteinatlas.org) was also used to verify the immunohistochemistry of hub genes.

### 2.5. Enrichment Analysis of Genes in Critical Modules and Hub Genes

g:Profiler (https://biit.cs.ut.ee/gprofiler/gost) is a web server for functional enrichment analysis and conversions of gene lists [14]. Gene Set Enrichment Analysis (GSEA) is a computational strategy that decides whether an a priori characterized set of genes appears measurably critical and has concordant contrasts between two biological states [15, 16]. We performed the Gene Ontology (GO) functional annotation analysis of the genes in the key modules and GSEA for the critical genes we chose with the assistance of g:Profiler and GSEA soft. *p* value < 0.05 was applied in the correction of false-positive results.

### 2.6. Evaluation of the Hub Genes and Establishment of a Better Signature for the PTC Survival Prediction

For the further study of the hub genes we got, we performed multivariate Cox regression analyses. We desired to clarify whether the hub genes could be applied to be the independent factors for the patient survival time. After that, we used the regression coefficients from the multivariate Cox regression analyses we did (*β*) and combined the hub gene expression levels to establish a new signature. Prognostic index (Pi) = (*β*∗expression level of ATRX) + (*β*∗expression level of BOD1L1) + (*β*∗expression level of CEP290) + (*β*∗expression level of DCAF16) + (*β*∗expression level of NEK1) [8]. According to the risk score, we divided patients into two groups: low risk and high risk. For validation of the signature, we did a series of visualization analyses. To evaluate the rationality of the new signature, we did a series of pictures including heat maps, survival plots, and survival curves according to the risk scores each patient got.

### 2.7. The Establishment of a Nomogram

Nomogram is an effective method for predicting the prognosis of cancer patients by simplifying the complex statistical prediction model into a profile chart for assessing the probability of OS in individual patients. In this study, we selected gender, age, T stage, bilateral position, and risk score as the independent factors to construct the nomogram. The nomogram signature could assess the survival possibility for PTC patients in the next two and three years.

## 3. Results

### 3.1. Weight Coexpression Network Construction

**Figure 1:**
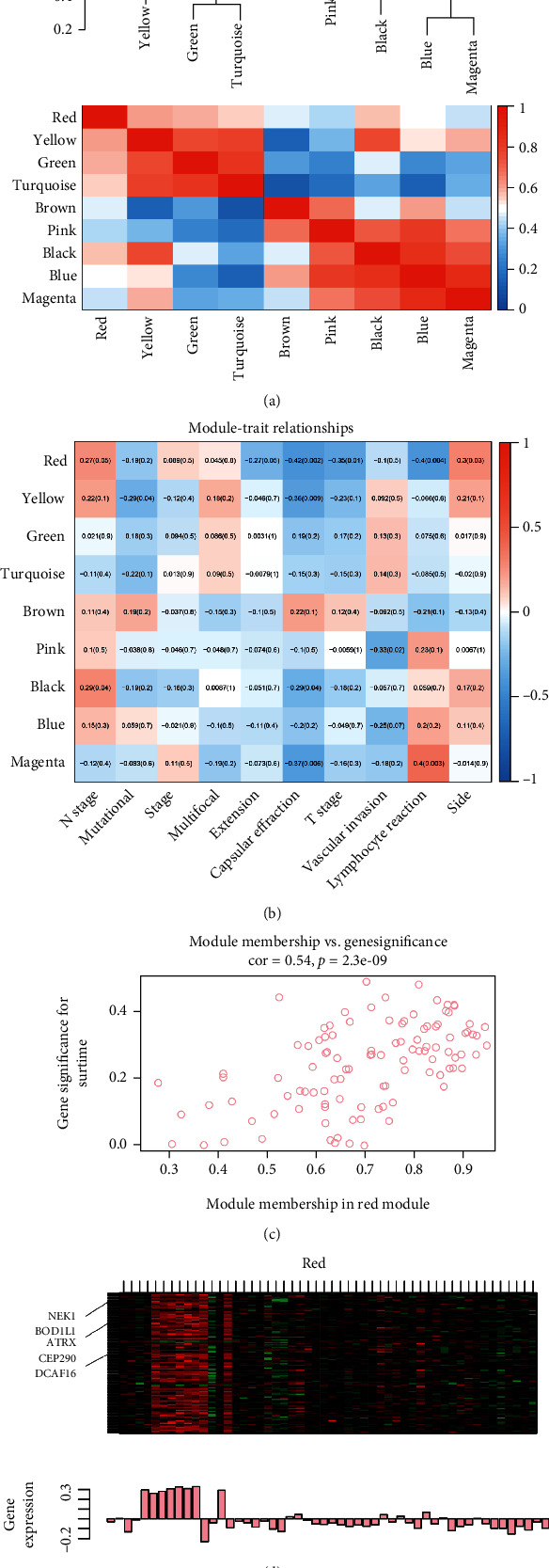
Identification of hub gene modules in papillary thyroid cancer patients: (a) the association heat map between different modules; (b) papillary thyroid cancer sample clusters; (c) the relationship between red module and survival time for PTC patients; (d) the gene expression characteristics in red modules.

4230 genes and 61 samples in GSE60542 were applied to construct the network (Supplementary figures 1A-B). The samples were clustered using the average linkage and Pearson correlation methods [13]. In this passage, we defined the soft-thresholding parameter *β* = 14 to build the free-scale system (Supplementary figure 1C). Scale-free *R*^2^ = 0.87 means the high quality of the network (Supplementary figure 1E). The way using average linkage hierarchical clustering identified ten modules (Supplementary figure 1D). For visualization, they were termed different colors (Figure 1(a)). The genes which cannot be clustered were put in the gray module. So we did not take this module for further study.

### 3.2. Key Module Identification

The red module significantly negatively correlated with the T stage (Figure 1(b)). What is more, the genes in this module are significantly positively related to survival time (Figure 1(c)). After evaluation of the genes' expression in the red module (Figure 1(d)), we selected the red module as a vital module with clinical significance.

### 3.3. Enrichment of Genes in the Red Modules

**Figure 2:**
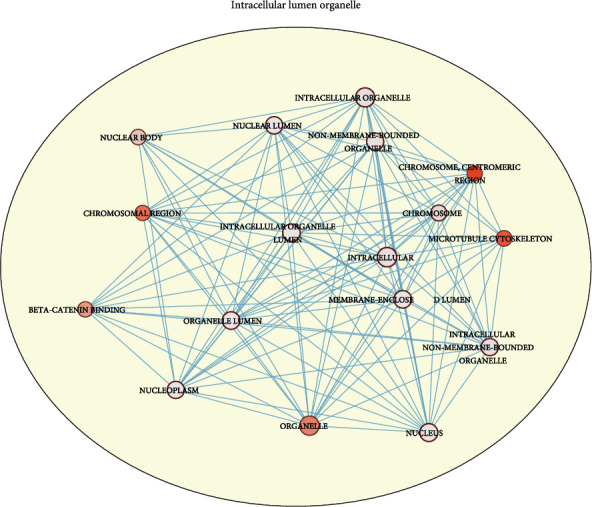
The enrichment results of the genes in the red module.

We underwent the functional annotation analysis of the genes in the red modules with g:Profiler (https://biit.cs.ut.ee/gprofiler/gost) [14] (Table. 1). Moreover, visualize the result using the Enrichment Map, a plugin of Cytoscape. The outcome demonstrated that the genes are mainly enriched in intracellular lumen organelle. They are mainly clustered at chromosomal and encoded construction protein (Figure 2). What we found indicated that the genes in the red module might play essential roles in cell growth, which exists in vast differences between tumor and normal tissues.

### 3.4. Hub Gene Identification and Validation

**Figure 3:**
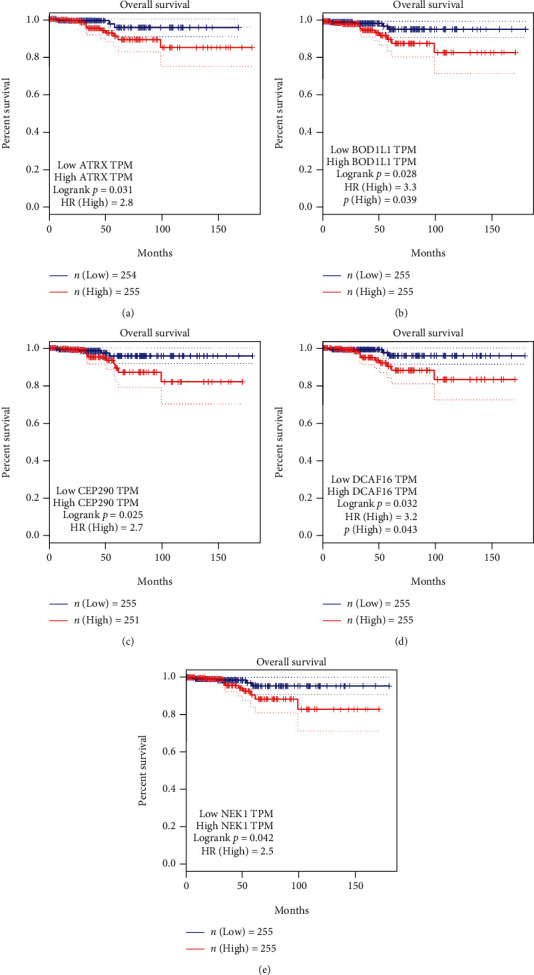
The Kaplan-Meier plots of PTC patients in TCGA with high and low expression of (a) ATRX, (b) BOD1L1, (c) CEP290, (d) DCAF16, and (e) NEK1.

Based on the cut-off standard mentioned previously (|M| > 0.8, |GS| > 0.2, and PPI degree score>1), 38 genes were selected. However, not all of them are meaningful in clinic work. We introduced the overall survival (OS) to text the clinic value of each gene. OS of the patients with thyroid cancer based on Kaplan Meier-plotter was from GEPIA (http://gepia.cancer-pku.cn/) [17], an online database based on TCGA. The patients with high expression of ATRX, BOD1L1, CEP290, DCAF16, and NEK1 have significantly shorter OS time, and they have a high hazard ratio (HR) (Figure 3).

**Figure 4:**
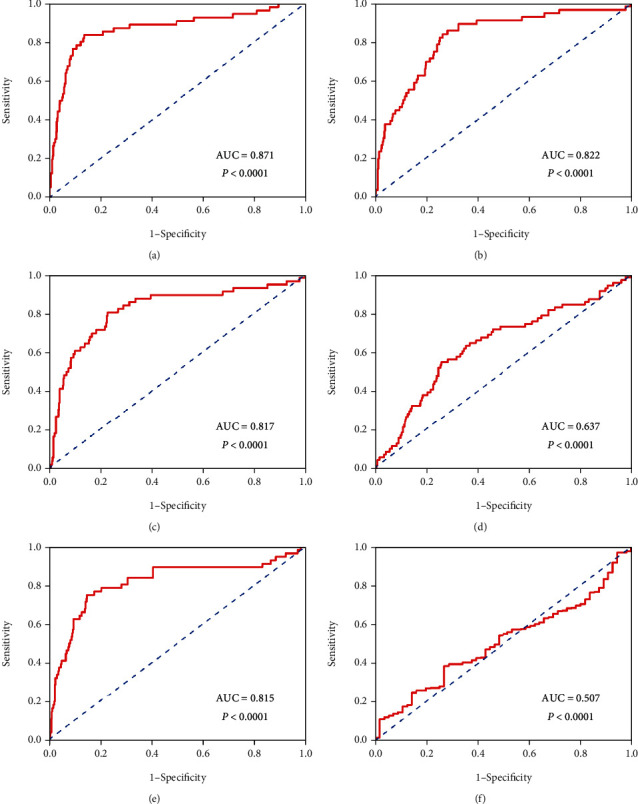
The ROC of different genes for PTC patients in TCGA: (a) NEK1, (b) DCAF16, (c) CEP290, (d) ATRX, (e) BOD1L1, and (f) BRAF.

**Figure 5:**
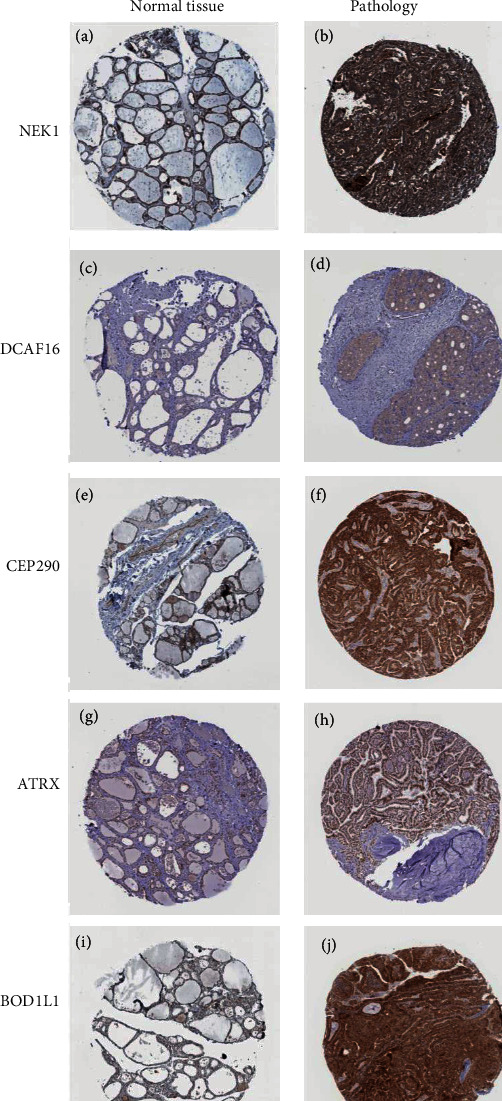
The immunohistochemical (IHC) pictures declared the different expressions between normal tissue and PTC: (a) NEK1 protein expression in normal tissue; (b) NEK1 protein expression in PTC; (c) DCAF16 protein expression in normal tissue; (d) DCAF16 protein expression in PTC; (e) CEP290 protein expression in normal tissue; (f) CEP290 protein expression in PTC; (g) ATRX protein expression in normal tissue; (h) ATRX protein expression in PTC; (i) BOD1L1 protein expression in normal tissue; (j) BOD1L1 protein expression in PTC.

Moreover, we validated these five genes' expression levels via TCGA. To evaluate the reliability of these five genes for prognosticating tumors, we created the ROC curve. The ROC curve demonstrated that ATRX, BOD1L1, CEP290, DCAF16, and NEK1 had enough efficiency for prognosticating PTC (Figures 4(a)–4(e)). What excites us is that these molecules performed better than PTC traditional diagnosis marker—BRAF, which indicated that these molecules might be potential clinical markers in further research (Figure 4(f)). The immunohistochemical (IHC) pictures from the Human Protein Atlas verified the higher expression of hub genes in PTC (Figure 5).

### 3.5. GSEA of Hub Genes and Correlation Analysis

**Figure 6:**
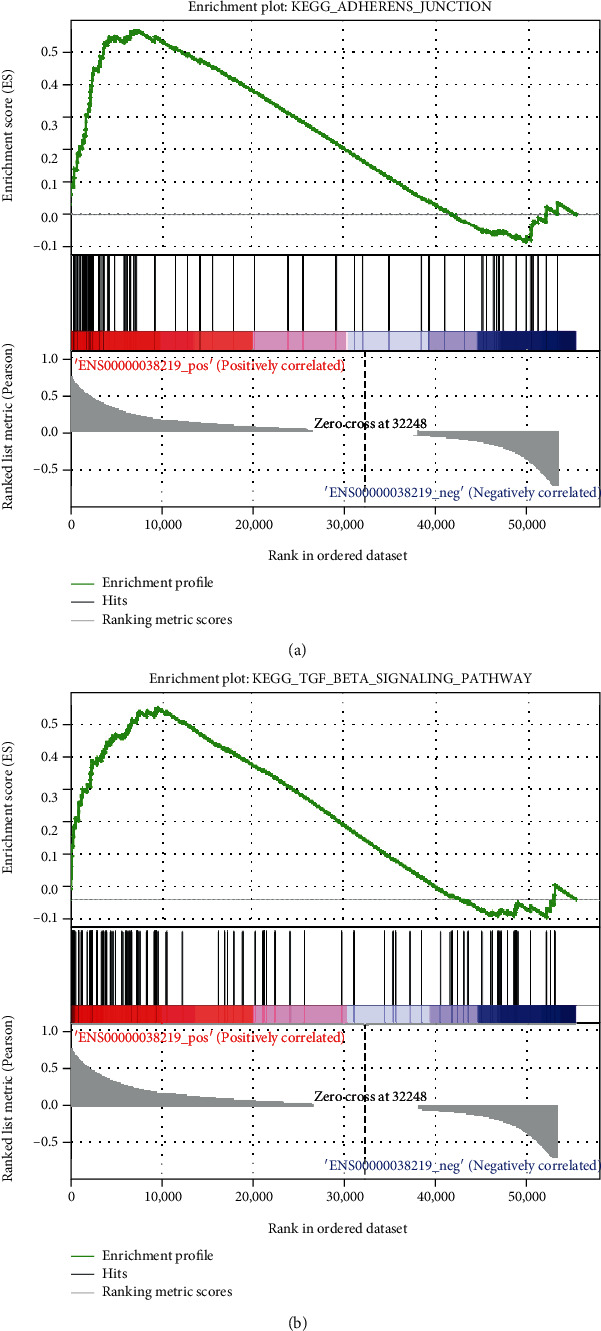
The BOD1L1 highly expressed PTC samples were significantly enriched in (a) TGF- (transforming growth factor-) *β* signaling pathway and (b) adherent junction pathways.

**Figure 7:**
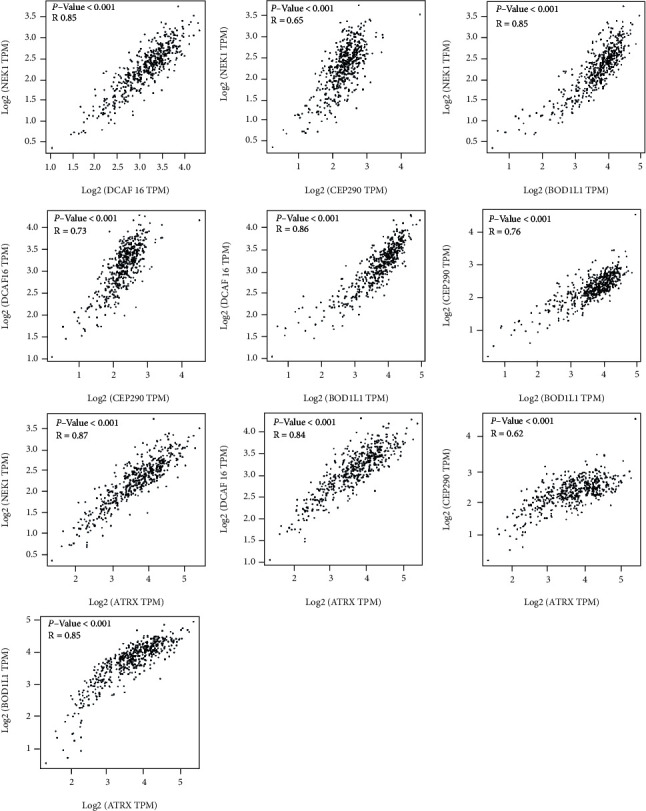
The close relationship between hub genes in this research.

After obtaining the “final” hub genes to explore the potential pathway they joined in the PTC, we performed the GSEA. We paid more attention to the pathway associated with tumors. What we found was that BOD1L1 got closely related to the TGF- (transforming growth factor-) beta signaling pathway and adherent junctions (FDR *q* − value < 0.05), which all facilitated the tumors' proliferation and transferal (Figures 6(a) and 6(b)). For more details about the GSEA results, please check Supplementary file 1 and Supplementary file 2. Then, we performed the correlation analysis and created the heat map with R soft and ggcorrplot package.  Figure 7 indicated that the hub genes we found are significantly related (*p* < 0.05).

### 3.6. Multivariate Cox Regression Analyses and Establishment of a New Signature for the PTC Survival Prediction

**Figure 8:**
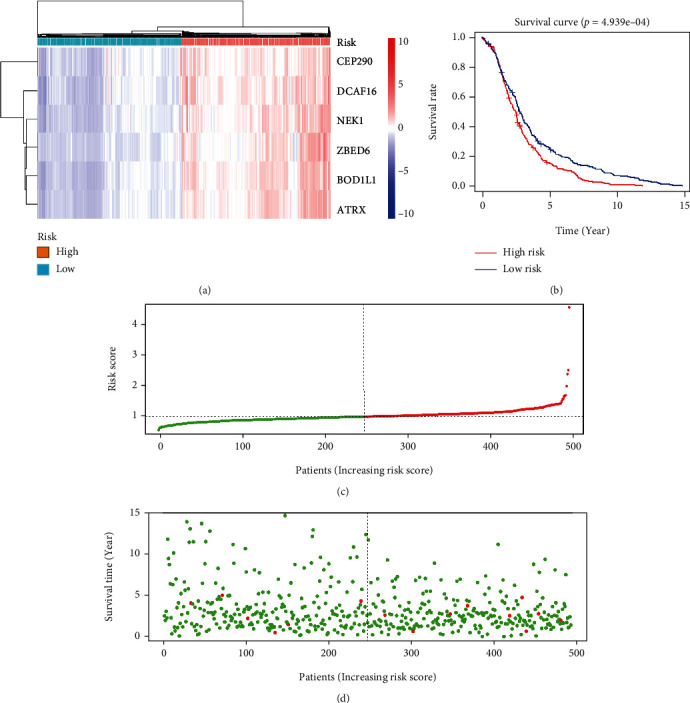
Characteristic analysis of the hub gene signature in TCGA cohort: (a) the heat map of hub genes in the high-risk score group and the low-risk score group; (b) the Kaplan-Meier plots of PTC patients in TCGA with high-risk and low-risk scores; (c) the distribution and median value of the risk scores in TCGA cohort; (d) the distributions of OS status and risk score in TCGA cohort.

In order to exploit the clinical predictive value of the model fully, we established a risk score signature based on the multivariate Cox regression analyses, as the above statement [8]. The new signature gave each PTC patient in TCGA a risk score. According to the median value of the risk score, the 410 PTC patients with enough follow-up information were divided into high-risk and low-risk groups. The heat map indicates the expression in high-risk and low-risk groups (Figure 8(a)). From the survival plots and survival curve, we found that the high-risk group in the PTC patients had more death cases and lower survival status (Figures 8(b)–8(d)).

### 3.7. Nomogram Curve

**Figure 9:**
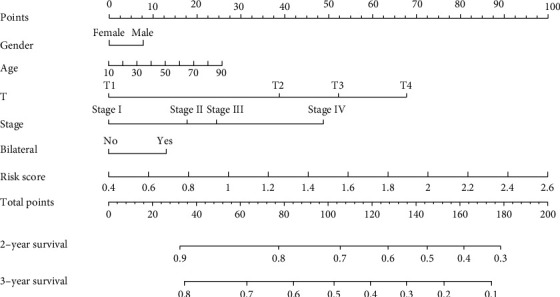
The clinical application of the hub gene signature by nomogram.

The nomogram shows every factor's impact on the OS of PTC in the next two and three years. Nomograms turned the patients' information into scores on a scale. We amounted to the total score and predicted the OS according to the total score (Figure 9).

## 4. Discussion

Thyroid cancer is a severe threat to the health of civilization in every age group. PTC is the most common malignant tumor of the thyroid gland [18]. It develops as an outcome of irregularities of specific genes that are generally liable for negative regulation in the expression of genes in cellular growth, proliferation, and differentiation. The TNM classification is a scientific system for classifying a malignancy [19]. T represents the tumor size and invasion ability. It is an essential phenotype for PTC, and the more advanced the T stage of the patients is, the worse their prognosis will be. It is an immediate requirement for biomarkers with high effectiveness. So we explored the potential biomarkers for PTC by combining the clinic information—T stage. In this study, we made use of the data from GSE60542 to screen potential biomarkers associated with PTC. We looked through TCGA database for PTC-related clinical and mRNA profiles for further verification.

WGCNA is an advanced data mining application that is widely used in tumors. It is unique for combining bioinformation with clinic information like TNM classification, stage, and mutation. Besides, hub genes within the modules are advanced mined to find genes that regulate tumor growth, proliferation, and differentiation. 4230 most variant genes and 53 PTC samples were incorporated into the study. We identified ten modules and found that the red module was highly negatively associated with the T stage. 38 genes satisfied the criteria in total. ATRX, BOD1L1, CEP290, DCAF16, and NEK1 were adversely related to the OS.

ATRX chromatin remodeler (ATRX) is a protein-coding gene. The protein encoded by it has an ATPase/helicase domain. The protein belongs to SWI/SNF complex chromatin remodeling proteins and performs cell cycle-dependent phosphorylation. ATRX loss-of-function mutations are associated with cancers that exhibit the ALT phenotype [20]. A study reports that ATRX expressed significantly higher in osteosarcoma compared with normal [21]. In thyroid carcinoma, some subtypes harbor mutations of SWI/SNF subunits, including ATRX, and the frequency of abnormal SWI/SNF complex is lower [22]. The result fitted ours well. ATRX might be a therapeutic target for further research.

Biorientation of chromosomes in cell division 1 like 1 (BOD1L1) is a protein coding gene. The protein encoded by BOD1L1 is a component of this fork protection pathway, which safeguards genome stability after replication stress [23]. Recently, scientists found that BOD1L1 is a replication fork protection factor that prevents the processing of stalled replication forks within the context of current knowledge of the replication fork proteasome [24]. Giurato et al. [25] reported that it modulates cancer cell proliferation and tumor growth, exerting an oncosuppressive role in breast cancer. In our study, the GSEA showed that BOD1L1 was significantly related to the TGF-*β* signaling pathway and adherent unction. TGF-*β* plays a role as a tumor suppressor at the early stage. However, it often changes the role of a tumor promoter during late progression [26]. Adherent junctions are essential for maintaining tissue architecture and cell polarity and can limit cell movement and proliferation, which is a tumor suppressor [27]. The downregulated expression of BOD1L1 might cause PTC through these pathways.

Centrosomal protein 290 (CEP290) encodes a large multidomain 290 kDa protein involved in cilia biogenesis and transport. The CEP290 protein constitutes an integral component of the transition zone (TZ) between the basal body and the ciliary axoneme, and it serves as a diffusion barrier for transport in and out of the cilium [28]. It is reported that CEP290 mutations cause a spectrum of ciliopathies from Leber congenital amaurosis type 10 to embryolethal Meckel syndrome [29]. Yu et al. [30] found that CEP290 is a susceptibility gene in hereditary nonpolyposis colorectal cancer.

Damage-Specific DNA Binding Protein 1 (DDB1) and Cullin-4 (CUL4) associated factor 16 (DCAF16) serve as substrate recognition components for the CUL4-DDB1 E3 ubiquitin-protein ligase complex, which mediates ubiquitination and proteasome-dependent degradation of nuclear proteins [31]. Liang et al. [32] reported that DCAF16 was expressed in human carcinomas, including adenocarcinoma, squamous cell carcinoma, and urothelial carcinoma. What they found indicated that DCAF16 can be an excellent prognostic gene for tumors.

NIMA-related kinase 1 (NEK1) is a serine/threonine kinase involved in cell cycle regulation but also appears to possess tyrosine kinase activity involved in DNA damage checkpoint control and proper DNA damage repair [33]. Zhu et al. [34] found that the abnormal regulation of NEK1 is a potential risk for colorectal tumorigenesis. Besides, Melo-Hanchuk et al. [35] reported that NEK1 expression was profoundly different when comparing malignant and benign thyroid tissue. Moreover, there is a difference in expression between the advanced and initial stages. The conclusion they drew was consistent with the results of this study.

WGCNA is an in-depth analysis of mRNA and microRNA massive datasets combining clinic phenotypes. It is widely used in multigene tumor research. We used WGCNA to construct a scale-free network with the GEO profile. The genes identified in various modules share similar expressions independent of the clinic information, and the measurement of the relationship between modules and clinic traits was done simultaneously. We picked up the red modules because it was significantly negatively associated with the T classification. GO analysis indicated that the genes in the module are mainly enriched in intracellular lumen organelles. After verification using TCGA database, we explored five genes as hub genes. They were ATRX, BOD1L1, CEP290, DCAF16, and NEK1. They have high coexpression, which suggests that the WGCNA worked well in dividing modules. These five genes had value for early diagnosis and represented different T stages of PTC. Finally, we used immunohistochemistry figures from HPA and next-generation sequencing in a laboratory to test the expression of the hub genes. The difference between the results of the online database and the tissues we collected might be owing to the limited scale of the samples in the laboratory. There needs further research about the left four hub genes. The results in this article might be applied in the clinic work and create a new thought for PTC research.

Though the hub genes were associated with the survival time according to the survival plots, the multivariate Cox regression analyses revealed that the single gene was not a perfect independent prognostic factor. This means that forecasting the prognostic outcome by just one gene was inaccurate. To make full use of the values of the final hub genes we found, we build a new signature for the PTC survival prediction with the support of the data from the multivariate Cox regression analyses. Through the new multivariate Cox regression analyses, we could announce that the risk scores ased on the novel signature were an ideal factor for prognostic. The survival time plots confirmed the same conclusion. At last, we did a quantitative nomogram to analyze ach factor's contributions to survival time. The model fits the general cognition of the clinic doctors.

## 5. Conclusion

PTC is a momentous kind of endocrine tumor, which possesses a high incidence rate. Many pieces of research have studied it using different methods. We performed the analysis with the help of WGCNA. However, through the multivariate Cox regression analyses, we concluded that selecting a single hub gene as a predictive factor for the prognosis of the PTC was not perfect. The new signature based on the results of the Cox regression analyses passed the examination and worked well as a predictive factor. The hub genes we found had some clinical significance and deserved more fundamental study. Our new signature could be applied in clinic work for its prognosis value.

## 6. Limitation

Morbidity in PTC results from a combination of factors. Only using data mining and basic experiment is biased or invalid. We still need more clinical practice to perfect our research.

## Figures and Tables

**Table 1:** The enrichment results of the red module genes.

Source	Term_name	Term_id	Adjusted_*p*_value
GO: MF.	Beta-catenin binding	GO:0008013	1.60*E*-02
G.O.:CC	Nucleoplasm	GO:0005654	8.90*E*-07
G.O.:CC	Nuclear lumen	GO:0031981	3.51*E*-06
G.O.:CC	Membrane-enclosed lumen	GO:0031974	7.04*E*-05
G.O.:CC	Organelle lumen	GO:0043233	7.04*E*-05
G.O.:CC	Intracellular organelle lumen	GO:0070013	7.04*E*-05
G.O.:CC	Nucleus	GO:0005634	2.13*E*-04
G.O.:CC	Intracellular	GO:0005622	2.52*E*-04
G.O.:CC	Intracellular non-membrane-bounded organelle	GO:0043232	4.16*E*-04
G.O.:CC	Non-membrane-bounded organelle	GO:0043228	4.40*E*-04
G.O.:CC	Intracellular organelle	GO:0043229	6.20*E*-04
G.O.:CC	Chromosome	GO:0005694	1.33*E*-03
G.O.:CC	Nuclear body	GO:0016604	5.49*E*-03
G.O.:CC	Organelle	GO:0043226	2.28*E*-02
G.O.:CC	Chromosomal region	GO:0098687	2.96*E*-02
G.O.:CC	Microtubule cytoskeleton	GO:0015630	3.93*E*-02
GO:CC	Chromosome, centromeric region	GO:0000775	4.97*E*-02

## Data Availability

All the data used in this research are from public datasets. All of them can be obtained with the guide mentioned in the method and result parts.

## Supplementary Materials

Supplementary Materials Supplementary figure 1: identification of prognostic genes in papillary thyroid cancer patients. (A) Clustering dendrogram of genome-wide genes in papillary thyroid cancer. (B) Papillary thyroid cancer sample clusters. (C) Analysis of the scale-free fit index for various soft-thresholding powers (*β*) and analysis of the mean connectivity for various soft-thresholding powers. (D) Clustering dendrogram of papillary thyroid cancer samples. (E) Checking the scale-free topology when *β* = 14. Supplementary file 1: the details about Figure 7(a). Supplementary file 2: the details about Figure 7(b).
