# Supplementary material for: Identification of Gene Coexpression Modules and Prognostic Genes Associated with Papillary Thyroid Cancer
Source: J Oncol. 2022 Sep 20;2022:9025198. doi: 10.1155/2022/9025198 (PMC9553521; doi:10.1155/2022/9025198)
Supplement: Supplementary Materials — Supplementary figure 1: identification of prognostic genes in papillary thyroid cancer patients. (A) Clustering dendrogram of genome-wide genes in papillary thyroid cancer. (B) Papillary thyroid cancer sample clusters. (C) Analysis of the scale-free fit index for various soft-thresholding powers (β) and analysis of the mean connectivity for various soft-thresholding powers. (D) Clustering dendrogram of papillary thyroid cancer samples. (E) Checking the scale-free topology when β = 14. Supplementary file 1: the details about Figure 7(a). Supplementary file 2: the details about Figure 7(b). [file 9025198.f1.zip › supplementary figures 1.docx]

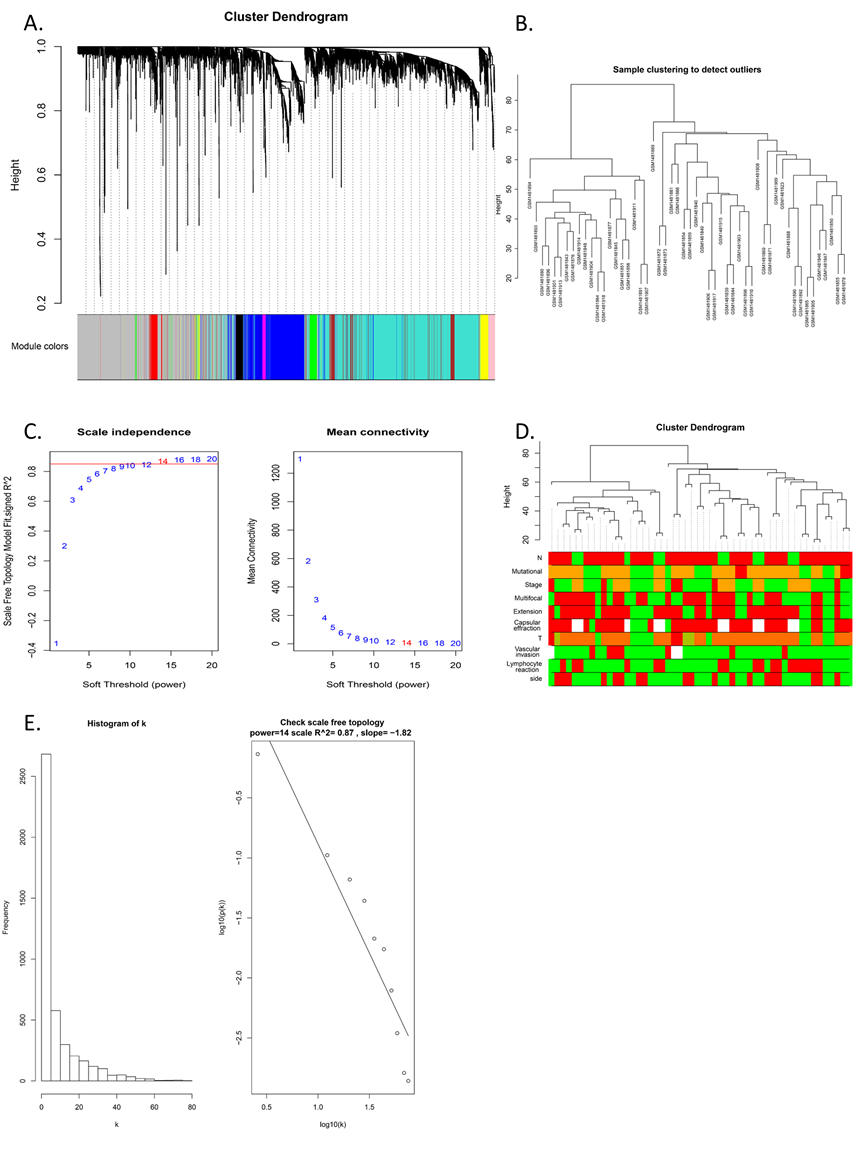


Supplementary figure 1. Identification of prognostic genes in papillary thyroid cancer patients (A) Clustering dendrogram of genome-wide genes in papillary thyroid cancer. (B) papillary thyroid cancer samples clusters. (C) Analysis of the scale-free fit index for various soft-thresholding powers (β) and analysis of the mean connectivity for various soft-thresholding powers. (D) Clustering dendrogram of papillary thyroid cancer samples. (E) Checking the scale-free topology when β = 14.
