# Supplementary material for: Identification of Gene Coexpression Modules and Prognostic Genes Associated with Papillary Thyroid Cancer
Source: J Oncol. 2022 Sep 20;2022:9025198. doi: 10.1155/2022/9025198 (PMC9553521; doi:10.1155/2022/9025198)
Supplement: Supplementary Materials — Supplementary figure 1: identification of prognostic genes in papillary thyroid cancer patients. (A) Clustering dendrogram of genome-wide genes in papillary thyroid cancer. (B) Papillary thyroid cancer sample clusters. (C) Analysis of the scale-free fit index for various soft-thresholding powers (β) and analysis of the mean connectivity for various soft-thresholding powers. (D) Clustering dendrogram of papillary thyroid cancer samples. (E) Checking the scale-free topology when β = 14. Supplementary file 1: the details about Figure 7(a). Supplementary file 2: the details about Figure 7(b). [file 9025198.f1.zip › supplementary files.docx]

Supplementary table 1 The details about Figure 7A

Dataset ExpData_collapsed_to_symbols.ENSG00000038219_profile_in_ExpData.cls

#ENSG00000038219

Phenotype ENSG00000038219_profile_in_ExpData.cls#ENSG00000038219

Upregulated in class ENSG00000038219_pos

GeneSet KEGG_ADHERENS_JUNCTION

Enrichment Score (ES) 0.5641985

Normalized Enrichment Score (NES) 1.8287425

Nominal p-value 0.0

FDR q-value 0.03437294

FWER p-Value 0.044

| **PROBE** | **DESCRIPTION (from dataset)** | **GENE SYMBOL** | **GENE_TITLE** | **RANK IN GENE LIST** | **RANK METRIC SCORE** | **RUNNING ES** | **CORE ENRICHMENT** |  |
| --- | --- | --- | --- | --- | --- | --- | --- | --- |
| 1 | [FER](https://ensembl.org/Search/Results?q=FER) | FER tyrosine kinase [Source:HGNC Symbol;Acc:HGNC:3655] |  |  | 105 | 0.814 | 0.0316 | Yes |
| 2 | [CREBBP](https://ensembl.org/Search/Results?q=CREBBP) | CREB binding protein [Source:HGNC Symbol;Acc:HGNC:2348] |  |  | 227 | 0.769 | 0.0610 | Yes |
| 3 | [EP300](https://ensembl.org/Search/Results?q=EP300) | E1A binding protein p300 [Source:HGNC Symbol;Acc:HGNC:3373] |  |  | 280 | 0.754 | 0.0911 | Yes |
| 4 | [TJP1](https://ensembl.org/Search/Results?q=TJP1) | tight junction protein 1 [Source:HGNC Symbol;Acc:HGNC:11827] |  |  | 628 | 0.680 | 0.1128 | Yes |
| 5 | [CTNND1](https://ensembl.org/Search/Results?q=CTNND1) | catenin delta 1 [Source:HGNC Symbol;Acc:HGNC:2515] |  |  | 662 | 0.676 | 0.1399 | Yes |
| 6 | [YES1](https://ensembl.org/Search/Results?q=YES1) | YES proto-oncogene 1, Src family tyrosine kinase [Source:HGNC Symbol;Acc:HGNC:12841] |  |  | 960 | 0.630 | 0.1605 | Yes |
| 7 | [FARP2](https://ensembl.org/Search/Results?q=FARP2) | FERM, ARH/RhoGEF and pleckstrin domain protein 2 [Source:HGNC Symbol;Acc:HGNC:16460] |  |  | 991 | 0.626 | 0.1857 | Yes |
| 8 | [AFDN](https://ensembl.org/Search/Results?q=AFDN) | afadin, adherens junction formation factor [Source:HGNC Symbol;Acc:HGNC:7137] |  |  | 1235 | 0.593 | 0.2056 | Yes |
| 9 | [MAP3K7](https://ensembl.org/Search/Results?q=MAP3K7) | mitogen-activated protein kinase kinase kinase 7 [Source:HGNC Symbol;Acc:HGNC:6859] |  |  | 1513 | 0.560 | 0.2236 | Yes |
| 10 | [CSNK2A1](https://ensembl.org/Search/Results?q=CSNK2A1) | casein kinase 2 alpha 1 [Source:HGNC Symbol;Acc:HGNC:2457] |  |  | 1713 | 0.540 | 0.2422 | Yes |
| 11 | [WASF2](https://ensembl.org/Search/Results?q=WASF2) | WASP family member 2 [Source:HGNC Symbol;Acc:HGNC:12733] |  |  | 1875 | 0.527 | 0.2609 | Yes |
| 12 | [MAPK1](https://ensembl.org/Search/Results?q=MAPK1) | mitogen-activated protein kinase 1 [Source:HGNC Symbol;Acc:HGNC:6871] |  |  | 1897 | 0.525 | 0.2821 | Yes |
| 13 | [WASL](https://ensembl.org/Search/Results?q=WASL) | WASP like actin nucleation promoting factor [Source:HGNC Symbol;Acc:HGNC:12735] |  |  | 1899 | 0.525 | 0.3037 | Yes |
| 14 | [IQGAP1](https://ensembl.org/Search/Results?q=IQGAP1) | IQ motif containing GTPase activating protein 1 [Source:HGNC Symbol;Acc:HGNC:6110] |  |  | 2153 | 0.503 | 0.3198 | Yes |
| 15 | [TGFBR2](https://ensembl.org/Search/Results?q=TGFBR2) | transforming growth factor beta receptor 2 [Source:HGNC Symbol;Acc:HGNC:11773] |  |  | 2277 | 0.491 | 0.3378 | Yes |
| 16 | [VCL](https://ensembl.org/Search/Results?q=VCL) | vinculin [Source:HGNC Symbol;Acc:HGNC:12665] |  |  | 2398 | 0.481 | 0.3554 | Yes |
| 17 | [SMAD4](https://ensembl.org/Search/Results?q=SMAD4) | SMAD family member 4 [Source:HGNC Symbol;Acc:HGNC:6770] |  |  | 2405 | 0.481 | 0.3750 | Yes |
| 18 | [EGFR](https://ensembl.org/Search/Results?q=EGFR) | epidermal growth factor receptor [Source:HGNC Symbol;Acc:HGNC:3236] |  |  | 2491 | 0.474 | 0.3930 | Yes |
| 19 | [PTPRJ](https://ensembl.org/Search/Results?q=PTPRJ) | protein tyrosine phosphatase receptor type J [Source:HGNC Symbol;Acc:HGNC:9673] |  |  | 2558 | 0.468 | 0.4110 | Yes |
| 20 | [SSX2IP](https://ensembl.org/Search/Results?q=SSX2IP) | SSX family member 2 interacting protein [Source:HGNC Symbol;Acc:HGNC:16509] |  |  | 2605 | 0.465 | 0.4293 | Yes |
| 21 | [LMO7](https://ensembl.org/Search/Results?q=LMO7) | LIM domain 7 [Source:HGNC Symbol;Acc:HGNC:6646] |  |  | 2732 | 0.456 | 0.4458 | Yes |
| 22 | [PTPN1](https://ensembl.org/Search/Results?q=PTPN1) | protein tyrosine phosphatase non-receptor type 1 [Source:HGNC Symbol;Acc:HGNC:9642] |  |  | 3280 | 0.418 | 0.4530 | Yes |
| 23 | [IGF1R](https://ensembl.org/Search/Results?q=IGF1R) | insulin like growth factor 1 receptor [Source:HGNC Symbol;Acc:HGNC:5465] |  |  | 3434 | 0.407 | 0.4670 | Yes |
| 24 | [CTNNB1](https://ensembl.org/Search/Results?q=CTNNB1) | catenin beta 1 [Source:HGNC Symbol;Acc:HGNC:2514] |  |  | 3461 | 0.405 | 0.4831 | Yes |
| 25 | [PTPRM](https://ensembl.org/Search/Results?q=PTPRM) | protein tyrosine phosphatase receptor type M [Source:HGNC Symbol;Acc:HGNC:9675] |  |  | 3783 | 0.384 | 0.4931 | Yes |
| 26 | [WASF3](https://ensembl.org/Search/Results?q=WASF3) | WASP family member 3 [Source:HGNC Symbol;Acc:HGNC:12734] |  |  | 3847 | 0.380 | 0.5076 | Yes |
| 27 | [SORBS1](https://ensembl.org/Search/Results?q=SORBS1) | sorbin and SH3 domain containing 1 [Source:HGNC Symbol;Acc:HGNC:14565] |  |  | 3880 | 0.378 | 0.5226 | Yes |
| 28 | [PTPRB](https://ensembl.org/Search/Results?q=PTPRB) | protein tyrosine phosphatase receptor type B [Source:HGNC Symbol;Acc:HGNC:9665] |  |  | 4294 | 0.356 | 0.5297 | Yes |
| 29 | [SMAD2](https://ensembl.org/Search/Results?q=SMAD2) | SMAD family member 2 [Source:HGNC Symbol;Acc:HGNC:6768] |  |  | 4441 | 0.349 | 0.5414 | Yes |
| 30 | [NECTIN3](https://ensembl.org/Search/Results?q=NECTIN3) | nectin cell adhesion molecule 3 [Source:HGNC Symbol;Acc:HGNC:17664] |  |  | 5059 | 0.318 | 0.5433 | Yes |
| 31 | [CDH1](https://ensembl.org/Search/Results?q=CDH1) | cadherin 1 [Source:HGNC Symbol;Acc:HGNC:1748] |  |  | 6067 | 0.277 | 0.5364 | Yes |
| 32 | [PTPRF](https://ensembl.org/Search/Results?q=PTPRF) | protein tyrosine phosphatase receptor type F [Source:HGNC Symbol;Acc:HGNC:9670] |  |  | 6343 | 0.266 | 0.5423 | Yes |
| 33 | [SMAD3](https://ensembl.org/Search/Results?q=SMAD3) | SMAD family member 3 [Source:HGNC Symbol;Acc:HGNC:6769] |  |  | 6695 | 0.254 | 0.5464 | Yes |
| 34 | [CTNNA3](https://ensembl.org/Search/Results?q=CTNNA3) | catenin alpha 3 [Source:HGNC Symbol;Acc:HGNC:2511] |  |  | 6747 | 0.253 | 0.5559 | Yes |
| 35 | [PARD3](https://ensembl.org/Search/Results?q=PARD3) | par-3 family cell polarity regulator [Source:HGNC Symbol;Acc:HGNC:16051] |  |  | 7101 | 0.242 | 0.5594 | Yes |
| 36 | [CTNNA1](https://ensembl.org/Search/Results?q=CTNNA1) | catenin alpha 1 [Source:HGNC Symbol;Acc:HGNC:2509] |  |  | 7368 | 0.233 | 0.5642 | Yes |
| 37 | [TGFBR1](https://ensembl.org/Search/Results?q=TGFBR1) | transforming growth factor beta receptor 1 [Source:HGNC Symbol;Acc:HGNC:11772] |  |  | 9372 | 0.182 | 0.5353 | No |
| 38 | [TCF7L2](https://ensembl.org/Search/Results?q=TCF7L2) | transcription factor 7 like 2 [Source:HGNC Symbol;Acc:HGNC:11641] |  |  | 9375 | 0.182 | 0.5428 | No |
| 39 | [NLK](https://ensembl.org/Search/Results?q=NLK) | nemo like kinase [Source:HGNC Symbol;Acc:HGNC:29858] |  |  | 11609 | 0.142 | 0.5081 | No |
| 40 | [MET](https://ensembl.org/Search/Results?q=MET) | MET proto-oncogene, receptor tyrosine kinase [Source:HGNC Symbol;Acc:HGNC:7029] |  |  | 12963 | 0.123 | 0.4885 | No |
| 41 | [WASF1](https://ensembl.org/Search/Results?q=WASF1) | WASP family member 1 [Source:HGNC Symbol;Acc:HGNC:12732] |  |  | 14291 | 0.107 | 0.4688 | No |
| 42 | [ACTN1](https://ensembl.org/Search/Results?q=ACTN1) | actinin alpha 1 [Source:HGNC Symbol;Acc:HGNC:163] |  |  | 14323 | 0.107 | 0.4726 | No |
| 43 | [FGFR1](https://ensembl.org/Search/Results?q=FGFR1) | fibroblast growth factor receptor 1 [Source:HGNC Symbol;Acc:HGNC:3688] |  |  | 15666 | 0.093 | 0.4521 | No |
| 44 | [ACTN4](https://ensembl.org/Search/Results?q=ACTN4) | actinin alpha 4 [Source:HGNC Symbol;Acc:HGNC:166] |  |  | 17924 | 0.073 | 0.4141 | No |
| 45 | [SNAI2](https://ensembl.org/Search/Results?q=SNAI2) | snail family transcriptional repressor 2 [Source:HGNC Symbol;Acc:HGNC:11094] |  |  | 20222 | 0.056 | 0.3747 | No |
| 46 | [INSR](https://ensembl.org/Search/Results?q=INSR) | insulin receptor [Source:HGNC Symbol;Acc:HGNC:6091] |  |  | 23859 | 0.034 | 0.3100 | No |
| 47 | [LEF1](https://ensembl.org/Search/Results?q=LEF1) | lymphoid enhancer binding factor 1 [Source:HGNC Symbol;Acc:HGNC:6551] |  |  | 25461 | 0.026 | 0.2820 | No |
| 48 | [SNAI1](https://ensembl.org/Search/Results?q=SNAI1) | snail family transcriptional repressor 1 [Source:HGNC Symbol;Acc:HGNC:11128] |  |  | 28989 | 0.007 | 0.2182 | No |
| 49 | [CDC42](https://ensembl.org/Search/Results?q=CDC42) | cell division cycle 42 [Source:HGNC Symbol;Acc:HGNC:1736] |  |  | 29119 | 0.006 | 0.2161 | No |
| 50 | [ACTN3](https://ensembl.org/Search/Results?q=ACTN3) | actinin alpha 3 (gene/pseudogene) [Source:HGNC Symbol;Acc:HGNC:165] |  |  | 31088 | -0.004 | 0.1806 | No |
| 51 | [CTNNA2](https://ensembl.org/Search/Results?q=CTNNA2) | catenin alpha 2 [Source:HGNC Symbol;Acc:HGNC:2510] |  |  | 31992 | -0.010 | 0.1646 | No |
| 52 | [TCF7L1](https://ensembl.org/Search/Results?q=TCF7L1) | transcription factor 7 like 1 [Source:HGNC Symbol;Acc:HGNC:11640] |  |  | 34864 | -0.026 | 0.1135 | No |
| 53 | [ACTN2](https://ensembl.org/Search/Results?q=ACTN2) | actinin alpha 2 [Source:HGNC Symbol;Acc:HGNC:164] |  |  | 38358 | -0.051 | 0.0521 | No |
| 54 | [TCF7](https://ensembl.org/Search/Results?q=TCF7) | transcription factor 7 [Source:HGNC Symbol;Acc:HGNC:11639] |  |  | 39174 | -0.058 | 0.0397 | No |
| 55 | [NECTIN1](https://ensembl.org/Search/Results?q=NECTIN1) | nectin cell adhesion molecule 1 [Source:HGNC Symbol;Acc:HGNC:9706] |  |  | 39195 | -0.058 | 0.0417 | No |
| 56 | [NECTIN4](https://ensembl.org/Search/Results?q=NECTIN4) | nectin cell adhesion molecule 4 [Source:HGNC Symbol;Acc:HGNC:19688] |  |  | 40920 | -0.075 | 0.0135 | No |
| 57 | [FYN](https://ensembl.org/Search/Results?q=FYN) | FYN proto-oncogene, Src family tyrosine kinase [Source:HGNC Symbol;Acc:HGNC:4037] |  |  | 43053 | -0.102 | -0.0211 | No |
| 58 | [ERBB2](https://ensembl.org/Search/Results?q=ERBB2) | erb-b2 receptor tyrosine kinase 2 [Source:HGNC Symbol;Acc:HGNC:3430] |  |  | 44995 | -0.138 | -0.0506 | No |
| 59 | [ACP1](https://ensembl.org/Search/Results?q=ACP1) | acid phosphatase 1 [Source:HGNC Symbol;Acc:HGNC:122] |  |  | 45460 | -0.150 | -0.0529 | No |
| 60 | [RAC2](https://ensembl.org/Search/Results?q=RAC2) | Rac family small GTPase 2 [Source:HGNC Symbol;Acc:HGNC:9802] |  |  | 46203 | -0.172 | -0.0593 | No |
| 61 | [SRC](https://ensembl.org/Search/Results?q=SRC) | SRC proto-oncogene, non-receptor tyrosine kinase [Source:HGNC Symbol;Acc:HGNC:11283] |  |  | 46542 | -0.183 | -0.0579 | No |
| 62 | [RHOA](https://ensembl.org/Search/Results?q=RHOA) | ras homolog family member A [Source:HGNC Symbol;Acc:HGNC:667] |  |  | 46785 | -0.192 | -0.0544 | No |
| 63 | [WAS](https://ensembl.org/Search/Results?q=WAS) | WASP actin nucleation promoting factor [Source:HGNC Symbol;Acc:HGNC:12731] |  |  | 47261 | -0.210 | -0.0544 | No |
| 64 | [CSNK2A2](https://ensembl.org/Search/Results?q=CSNK2A2) | casein kinase 2 alpha 2 [Source:HGNC Symbol;Acc:HGNC:2459] |  |  | 48638 | -0.277 | -0.0680 | No |
| 65 | [BAIAP2](https://ensembl.org/Search/Results?q=BAIAP2) | BAI1 associated protein 2 [Source:HGNC Symbol;Acc:HGNC:947] |  |  | 49716 | -0.356 | -0.0729 | No |
| 66 | [NECTIN2](https://ensembl.org/Search/Results?q=NECTIN2) | nectin cell adhesion molecule 2 [Source:HGNC Symbol;Acc:HGNC:9707] |  |  | 50242 | -0.404 | -0.0658 | No |
| 67 | [ACTB](https://ensembl.org/Search/Results?q=ACTB) | actin beta [Source:HGNC Symbol;Acc:HGNC:132] |  |  | 50291 | -0.408 | -0.0499 | No |
| 68 | [RAC3](https://ensembl.org/Search/Results?q=RAC3) | Rac family small GTPase 3 [Source:HGNC Symbol;Acc:HGNC:9803] |  |  | 50306 | -0.409 | -0.0333 | No |
| 69 | [ACTG1](https://ensembl.org/Search/Results?q=ACTG1) | actin gamma 1 [Source:HGNC Symbol;Acc:HGNC:144] |  |  | 50502 | -0.427 | -0.0193 | No |
| 70 | [PTPN6](https://ensembl.org/Search/Results?q=PTPN6) | protein tyrosine phosphatase non-receptor type 6 [Source:HGNC Symbol;Acc:HGNC:9658] |  |  | 51042 | -0.486 | -0.0091 | No |
| 71 | [RAC1](https://ensembl.org/Search/Results?q=RAC1) | Rac family small GTPase 1 [Source:HGNC Symbol;Acc:HGNC:9801] |  |  | 51872 | -0.592 | 0.0002 | No |
| 72 | [MAPK3](https://ensembl.org/Search/Results?q=MAPK3) | mitogen-activated protein kinase 3 [Source:HGNC Symbol;Acc:HGNC:6877] |  |  | 51913 | -0.600 | 0.0241 | No |
| 73 | [CSNK2B](https://ensembl.org/Search/Results?q=CSNK2B) | casein kinase 2 beta [Source:HGNC Symbol;Acc:HGNC:2460] |  |  | 53105 | -0.830 | 0.0366 | No |

Supplementary table 2 The details about Figure 7B

Dataset ExpData_collapsed_to_symbols.ENSG00000038219_profile_in_ExpData.cls

#ENSG00000038219

Phenotype ENSG00000038219_profile_in_ExpData.cls#ENSG00000038219

Upregulated in class ENSG00000038219_pos

GeneSet KEGG_TGF_BETA_SIGNALING_PATHWAY

Enrichment Score (ES) 0.53826815

Normalized Enrichment Score (NES) 1.7662284

Nominal p-value 0.0

FDR q-value 0.045586843

FWER p-Value 0.11

| **PROBE** | **DESCRIPTION (from dataset)** | **GENE SYMBOL** | **GENE_TITLE** | **RANK IN GENE LIST** | **RANK METRIC SCORE** | **RUNNING ES** | **CORE ENRICHMENT** |  |
| --- | --- | --- | --- | --- | --- | --- | --- | --- |
| 1 | [RBL2](https://ensembl.org/Search/Results?q=RBL2) | RB transcriptional corepressor like 2 [Source:HGNC Symbol;Acc:HGNC:9894] |  |  | 66 | 0.834 | 0.0321 | Yes |
| 2 | [ROCK1](https://ensembl.org/Search/Results?q=ROCK1) | Rho associated coiled-coil containing protein kinase 1 [Source:HGNC Symbol;Acc:HGNC:10251] |  |  | 161 | 0.791 | 0.0620 | Yes |
| 3 | [CREBBP](https://ensembl.org/Search/Results?q=CREBBP) | CREB binding protein [Source:HGNC Symbol;Acc:HGNC:2348] |  |  | 227 | 0.769 | 0.0915 | Yes |
| 4 | [SMAD1](https://ensembl.org/Search/Results?q=SMAD1) | SMAD family member 1 [Source:HGNC Symbol;Acc:HGNC:6767] |  |  | 243 | 0.763 | 0.1218 | Yes |
| 5 | [EP300](https://ensembl.org/Search/Results?q=EP300) | E1A binding protein p300 [Source:HGNC Symbol;Acc:HGNC:3373] |  |  | 280 | 0.754 | 0.1513 | Yes |
| 6 | [BMPR2](https://ensembl.org/Search/Results?q=BMPR2) | bone morphogenetic protein receptor type 2 [Source:HGNC Symbol;Acc:HGNC:1078] |  |  | 398 | 0.726 | 0.1781 | Yes |
| 7 | [ROCK2](https://ensembl.org/Search/Results?q=ROCK2) | Rho associated coiled-coil containing protein kinase 2 [Source:HGNC Symbol;Acc:HGNC:10252] |  |  | 475 | 0.711 | 0.2052 | Yes |
| 8 | [SMAD5](https://ensembl.org/Search/Results?q=SMAD5) | SMAD family member 5 [Source:HGNC Symbol;Acc:HGNC:6771] |  |  | 630 | 0.679 | 0.2295 | Yes |
| 9 | [ZFYVE16](https://ensembl.org/Search/Results?q=ZFYVE16) | zinc finger FYVE-type containing 16 [Source:HGNC Symbol;Acc:HGNC:20756] |  |  | 999 | 0.625 | 0.2478 | Yes |
| 10 | [SP1](https://ensembl.org/Search/Results?q=SP1) | Sp1 transcription factor [Source:HGNC Symbol;Acc:HGNC:11205] |  |  | 1008 | 0.624 | 0.2726 | Yes |
| 11 | [RPS6KB1](https://ensembl.org/Search/Results?q=RPS6KB1) | ribosomal protein S6 kinase B1 [Source:HGNC Symbol;Acc:HGNC:10436] |  |  | 1259 | 0.590 | 0.2916 | Yes |
| 12 | [ZFYVE9](https://ensembl.org/Search/Results?q=ZFYVE9) | zinc finger FYVE-type containing 9 [Source:HGNC Symbol;Acc:HGNC:6775] |  |  | 1411 | 0.572 | 0.3117 | Yes |
| 13 | [MAPK1](https://ensembl.org/Search/Results?q=MAPK1) | mitogen-activated protein kinase 1 [Source:HGNC Symbol;Acc:HGNC:6871] |  |  | 1897 | 0.525 | 0.3239 | Yes |
| 14 | [TFDP1](https://ensembl.org/Search/Results?q=TFDP1) | transcription factor Dp-1 [Source:HGNC Symbol;Acc:HGNC:11749] |  |  | 2255 | 0.493 | 0.3371 | Yes |
| 15 | [TGFBR2](https://ensembl.org/Search/Results?q=TGFBR2) | transforming growth factor beta receptor 2 [Source:HGNC Symbol;Acc:HGNC:11773] |  |  | 2277 | 0.491 | 0.3563 | Yes |
| 16 | [SMAD4](https://ensembl.org/Search/Results?q=SMAD4) | SMAD family member 4 [Source:HGNC Symbol;Acc:HGNC:6770] |  |  | 2405 | 0.481 | 0.3732 | Yes |
| 17 | [SMURF1](https://ensembl.org/Search/Results?q=SMURF1) | SMAD specific E3 ubiquitin protein ligase 1 [Source:HGNC Symbol;Acc:HGNC:16807] |  |  | 2474 | 0.475 | 0.3910 | Yes |
| 18 | [ACVR2A](https://ensembl.org/Search/Results?q=ACVR2A) | activin A receptor type 2A [Source:HGNC Symbol;Acc:HGNC:173] |  |  | 3008 | 0.436 | 0.3987 | Yes |
| 19 | [SMURF2](https://ensembl.org/Search/Results?q=SMURF2) | SMAD specific E3 ubiquitin protein ligase 2 [Source:HGNC Symbol;Acc:HGNC:16809] |  |  | 3477 | 0.404 | 0.4064 | Yes |
| 20 | [BMPR1A](https://ensembl.org/Search/Results?q=BMPR1A) | bone morphogenetic protein receptor type 1A [Source:HGNC Symbol;Acc:HGNC:1076] |  |  | 3686 | 0.390 | 0.4182 | Yes |
| 21 | [TGFB2](https://ensembl.org/Search/Results?q=TGFB2) | transforming growth factor beta 2 [Source:HGNC Symbol;Acc:HGNC:11768] |  |  | 3906 | 0.377 | 0.4293 | Yes |
| 22 | [SMAD9](https://ensembl.org/Search/Results?q=SMAD9) | SMAD family member 9 [Source:HGNC Symbol;Acc:HGNC:6774] |  |  | 4054 | 0.369 | 0.4413 | Yes |
| 23 | [SMAD2](https://ensembl.org/Search/Results?q=SMAD2) | SMAD family member 2 [Source:HGNC Symbol;Acc:HGNC:6768] |  |  | 4441 | 0.349 | 0.4483 | Yes |
| 24 | [ACVR2B](https://ensembl.org/Search/Results?q=ACVR2B) | activin A receptor type 2B [Source:HGNC Symbol;Acc:HGNC:174] |  |  | 4668 | 0.336 | 0.4576 | Yes |
| 25 | [RBL1](https://ensembl.org/Search/Results?q=RBL1) | RB transcriptional corepressor like 1 [Source:HGNC Symbol;Acc:HGNC:9893] |  |  | 4980 | 0.322 | 0.4648 | Yes |
| 26 | [ACVR1](https://ensembl.org/Search/Results?q=ACVR1) | activin A receptor type 1 [Source:HGNC Symbol;Acc:HGNC:171] |  |  | 5712 | 0.291 | 0.4631 | Yes |
| 27 | [ACVR1C](https://ensembl.org/Search/Results?q=ACVR1C) | activin A receptor type 1C [Source:HGNC Symbol;Acc:HGNC:18123] |  |  | 6046 | 0.277 | 0.4682 | Yes |
| 28 | [BMPR1B](https://ensembl.org/Search/Results?q=BMPR1B) | bone morphogenetic protein receptor type 1B [Source:HGNC Symbol;Acc:HGNC:1077] |  |  | 6219 | 0.271 | 0.4759 | Yes |
| 29 | [THBS1](https://ensembl.org/Search/Results?q=THBS1) | thrombospondin 1 [Source:HGNC Symbol;Acc:HGNC:11785] |  |  | 6392 | 0.265 | 0.4833 | Yes |
| 30 | [INHBC](https://ensembl.org/Search/Results?q=INHBC) | inhibin subunit beta C [Source:HGNC Symbol;Acc:HGNC:6068] |  |  | 6534 | 0.260 | 0.4911 | Yes |
| 31 | [SMAD3](https://ensembl.org/Search/Results?q=SMAD3) | SMAD family member 3 [Source:HGNC Symbol;Acc:HGNC:6769] |  |  | 6695 | 0.254 | 0.4984 | Yes |
| 32 | [PPP2R1B](https://ensembl.org/Search/Results?q=PPP2R1B) | protein phosphatase 2 scaffold subunit Abeta [Source:HGNC Symbol;Acc:HGNC:9303] |  |  | 6764 | 0.252 | 0.5072 | Yes |
| 33 | [GDF7](https://ensembl.org/Search/Results?q=GDF7) | growth differentiation factor 7 [Source:HGNC Symbol;Acc:HGNC:4222] |  |  | 7250 | 0.237 | 0.5079 | Yes |
| 34 | [E2F5](https://ensembl.org/Search/Results?q=E2F5) | E2F transcription factor 5 [Source:HGNC Symbol;Acc:HGNC:3119] |  |  | 7373 | 0.233 | 0.5150 | Yes |
| 35 | [BMP8B](https://ensembl.org/Search/Results?q=BMP8B) | bone morphogenetic protein 8b [Source:HGNC Symbol;Acc:HGNC:1075] |  |  | 7420 | 0.232 | 0.5234 | Yes |
| 36 | [BMP6](https://ensembl.org/Search/Results?q=BMP6) | bone morphogenetic protein 6 [Source:HGNC Symbol;Acc:HGNC:1073] |  |  | 7601 | 0.227 | 0.5292 | Yes |
| 37 | [PPP2CB](https://ensembl.org/Search/Results?q=PPP2CB) | protein phosphatase 2 catalytic subunit beta [Source:HGNC Symbol;Acc:HGNC:9300] |  |  | 8385 | 0.206 | 0.5232 | Yes |
| 38 | [SMAD7](https://ensembl.org/Search/Results?q=SMAD7) | SMAD family member 7 [Source:HGNC Symbol;Acc:HGNC:6773] |  |  | 8432 | 0.204 | 0.5305 | Yes |
| 39 | [MYC](https://ensembl.org/Search/Results?q=MYC) | MYC proto-oncogene, bHLH transcription factor [Source:HGNC Symbol;Acc:HGNC:7553] |  |  | 9227 | 0.185 | 0.5235 | Yes |
| 40 | [BMP8A](https://ensembl.org/Search/Results?q=BMP8A) | bone morphogenetic protein 8a [Source:HGNC Symbol;Acc:HGNC:21650] |  |  | 9352 | 0.183 | 0.5286 | Yes |
| 41 | [TGFBR1](https://ensembl.org/Search/Results?q=TGFBR1) | transforming growth factor beta receptor 1 [Source:HGNC Symbol;Acc:HGNC:11772] |  |  | 9372 | 0.182 | 0.5355 | Yes |
| 42 | [INHBA](https://ensembl.org/Search/Results?q=INHBA) | inhibin subunit beta A [Source:HGNC Symbol;Acc:HGNC:6066] |  |  | 9610 | 0.177 | 0.5383 | Yes |
| 43 | [BMP2](https://ensembl.org/Search/Results?q=BMP2) | bone morphogenetic protein 2 [Source:HGNC Symbol;Acc:HGNC:1069] |  |  | 10548 | 0.160 | 0.5276 | No |
| 44 | [CUL1](https://ensembl.org/Search/Results?q=CUL1) | cullin 1 [Source:HGNC Symbol;Acc:HGNC:2551] |  |  | 12303 | 0.133 | 0.5011 | No |
| 45 | [LTBP1](https://ensembl.org/Search/Results?q=LTBP1) | latent transforming growth factor beta binding protein 1 [Source:HGNC Symbol;Acc:HGNC:6714] |  |  | 16181 | 0.088 | 0.4341 | No |
| 46 | [GDF6](https://ensembl.org/Search/Results?q=GDF6) | growth differentiation factor 6 [Source:HGNC Symbol;Acc:HGNC:4221] |  |  | 16850 | 0.082 | 0.4253 | No |
| 47 | [CDKN2B](https://ensembl.org/Search/Results?q=CDKN2B) | cyclin dependent kinase inhibitor 2B [Source:HGNC Symbol;Acc:HGNC:1788] |  |  | 17126 | 0.080 | 0.4235 | No |
| 48 | [THBS2](https://ensembl.org/Search/Results?q=THBS2) | thrombospondin 2 [Source:HGNC Symbol;Acc:HGNC:11786] |  |  | 17856 | 0.073 | 0.4131 | No |
| 49 | [THBS4](https://ensembl.org/Search/Results?q=THBS4) | thrombospondin 4 [Source:HGNC Symbol;Acc:HGNC:11788] |  |  | 18810 | 0.066 | 0.3985 | No |
| 50 | [PITX2](https://ensembl.org/Search/Results?q=PITX2) | paired like homeodomain 2 [Source:HGNC Symbol;Acc:HGNC:9005] |  |  | 20159 | 0.057 | 0.3763 | No |
| 51 | [NODAL](https://ensembl.org/Search/Results?q=NODAL) | nodal growth differentiation factor [Source:HGNC Symbol;Acc:HGNC:7865] |  |  | 21039 | 0.051 | 0.3623 | No |
| 52 | [ID4](https://ensembl.org/Search/Results?q=ID4) | inhibitor of DNA binding 4, HLH protein [Source:HGNC Symbol;Acc:HGNC:5363] |  |  | 21136 | 0.051 | 0.3626 | No |
| 53 | [IFNG](https://ensembl.org/Search/Results?q=IFNG) | interferon gamma [Source:HGNC Symbol;Acc:HGNC:5438] |  |  | 21423 | 0.049 | 0.3594 | No |
| 54 | [BMP5](https://ensembl.org/Search/Results?q=BMP5) | bone morphogenetic protein 5 [Source:HGNC Symbol;Acc:HGNC:1072] |  |  | 22368 | 0.043 | 0.3439 | No |
| 55 | [DCN](https://ensembl.org/Search/Results?q=DCN) | decorin [Source:HGNC Symbol;Acc:HGNC:2705] |  |  | 24003 | 0.033 | 0.3156 | No |
| 56 | [TGFB3](https://ensembl.org/Search/Results?q=TGFB3) | transforming growth factor beta 3 [Source:HGNC Symbol;Acc:HGNC:11769] |  |  | 25543 | 0.025 | 0.2886 | No |
| 57 | [TNF](https://ensembl.org/Search/Results?q=TNF) | tumor necrosis factor [Source:HGNC Symbol;Acc:HGNC:11892] |  |  | 29684 | 0.003 | 0.2135 | No |
| 58 | [FST](https://ensembl.org/Search/Results?q=FST) | follistatin [Source:HGNC Symbol;Acc:HGNC:3971] |  |  | 30986 | -0.004 | 0.1900 | No |
| 59 | [BMP4](https://ensembl.org/Search/Results?q=BMP4) | bone morphogenetic protein 4 [Source:HGNC Symbol;Acc:HGNC:1071] |  |  | 34351 | -0.023 | 0.1298 | No |
| 60 | [INHBB](https://ensembl.org/Search/Results?q=INHBB) | inhibin subunit beta B [Source:HGNC Symbol;Acc:HGNC:6067] |  |  | 35237 | -0.028 | 0.1149 | No |
| 61 | [SMAD6](https://ensembl.org/Search/Results?q=SMAD6) | SMAD family member 6 [Source:HGNC Symbol;Acc:HGNC:6772] |  |  | 35552 | -0.030 | 0.1104 | No |
| 62 | [BMP7](https://ensembl.org/Search/Results?q=BMP7) | bone morphogenetic protein 7 [Source:HGNC Symbol;Acc:HGNC:1074] |  |  | 37076 | -0.041 | 0.0843 | No |
| 63 | [ID2](https://ensembl.org/Search/Results?q=ID2) | inhibitor of DNA binding 2 [Source:HGNC Symbol;Acc:HGNC:5361] |  |  | 38341 | -0.051 | 0.0634 | No |
| 64 | [COMP](https://ensembl.org/Search/Results?q=COMP) | cartilage oligomeric matrix protein [Source:HGNC Symbol;Acc:HGNC:2227] |  |  | 40391 | -0.069 | 0.0289 | No |
| 65 | [NOG](https://ensembl.org/Search/Results?q=NOG) | noggin [Source:HGNC Symbol;Acc:HGNC:7866] |  |  | 41496 | -0.081 | 0.0121 | No |
| 66 | [LEFTY1](https://ensembl.org/Search/Results?q=LEFTY1) | left-right determination factor 1 [Source:HGNC Symbol;Acc:HGNC:6552] |  |  | 41689 | -0.083 | 0.0119 | No |
| 67 | [AMHR2](https://ensembl.org/Search/Results?q=AMHR2) | anti-Mullerian hormone receptor type 2 [Source:HGNC Symbol;Acc:HGNC:465] |  |  | 42214 | -0.089 | 0.0060 | No |
| 68 | [LEFTY2](https://ensembl.org/Search/Results?q=LEFTY2) | left-right determination factor 2 [Source:HGNC Symbol;Acc:HGNC:3122] |  |  | 42924 | -0.100 | -0.0029 | No |
| 69 | [INHBE](https://ensembl.org/Search/Results?q=INHBE) | inhibin subunit beta E [Source:HGNC Symbol;Acc:HGNC:24029] |  |  | 43368 | -0.107 | -0.0067 | No |
| 70 | [PPP2CA](https://ensembl.org/Search/Results?q=PPP2CA) | protein phosphatase 2 catalytic subunit alpha [Source:HGNC Symbol;Acc:HGNC:9299] |  |  | 44914 | -0.137 | -0.0293 | No |
| 71 | [ID3](https://ensembl.org/Search/Results?q=ID3) | inhibitor of DNA binding 3, HLH protein [Source:HGNC Symbol;Acc:HGNC:5362] |  |  | 45845 | -0.161 | -0.0397 | No |
| 72 | [CHRD](https://ensembl.org/Search/Results?q=CHRD) | chordin [Source:HGNC Symbol;Acc:HGNC:1949] |  |  | 46062 | -0.168 | -0.0369 | No |
| 73 | [ACVRL1](https://ensembl.org/Search/Results?q=ACVRL1) | activin A receptor like type 1 [Source:HGNC Symbol;Acc:HGNC:175] |  |  | 46644 | -0.187 | -0.0400 | No |
| 74 | [RHOA](https://ensembl.org/Search/Results?q=RHOA) | ras homolog family member A [Source:HGNC Symbol;Acc:HGNC:667] |  |  | 46785 | -0.192 | -0.0349 | No |
| 75 | [SKP1](https://ensembl.org/Search/Results?q=SKP1) | S-phase kinase associated protein 1 [Source:HGNC Symbol;Acc:HGNC:10899] |  |  | 46972 | -0.199 | -0.0303 | No |
| 76 | [GDF5](https://ensembl.org/Search/Results?q=GDF5) | growth differentiation factor 5 [Source:HGNC Symbol;Acc:HGNC:4220] |  |  | 47684 | -0.229 | -0.0341 | No |
| 77 | [TGFB1](https://ensembl.org/Search/Results?q=TGFB1) | transforming growth factor beta 1 [Source:HGNC Symbol;Acc:HGNC:11766] |  |  | 48407 | -0.266 | -0.0366 | No |
| 78 | [ID1](https://ensembl.org/Search/Results?q=ID1) | inhibitor of DNA binding 1, HLH protein [Source:HGNC Symbol;Acc:HGNC:5360] |  |  | 48536 | -0.272 | -0.0280 | No |
| 79 | [THBS3](https://ensembl.org/Search/Results?q=THBS3) | thrombospondin 3 [Source:HGNC Symbol;Acc:HGNC:11787] |  |  | 48611 | -0.276 | -0.0184 | No |
| 80 | [AMH](https://ensembl.org/Search/Results?q=AMH) | anti-Mullerian hormone [Source:HGNC Symbol;Acc:HGNC:464] |  |  | 48803 | -0.288 | -0.0103 | No |
| 81 | [E2F4](https://ensembl.org/Search/Results?q=E2F4) | E2F transcription factor 4 [Source:HGNC Symbol;Acc:HGNC:3118] |  |  | 50189 | -0.399 | -0.0195 | No |
| 82 | [MAPK3](https://ensembl.org/Search/Results?q=MAPK3) | mitogen-activated protein kinase 3 [Source:HGNC Symbol;Acc:HGNC:6877] |  |  | 51913 | -0.600 | -0.0269 | No |
| 83 | [RPS6KB2](https://ensembl.org/Search/Results?q=RPS6KB2) | ribosomal protein S6 kinase B2 [Source:HGNC Symbol;Acc:HGNC:10437] |  |  | 52342 | -0.658 | -0.0084 | No |
| 84 | [RBX1](https://ensembl.org/Search/Results?q=RBX1) | ring-box 1 [Source:HGNC Symbol;Acc:HGNC:9928] |  |  | 52728 | -0.727 | 0.0137 | No |
| 85 | [PPP2R1A](https://ensembl.org/Search/Results?q=PPP2R1A) | protein phosphatase 2 scaffold subunit Aalpha [Source:HGNC Symbol;Acc:HGNC:9302] |  |  | 52812 | -0.745 | 0.0419 | No |
